# Marital Status and Gender Differences in End-of-Life Planning

**DOI:** 10.1093/geroni/igaa057.1465

**Published:** 2020-12-16

**Authors:** Lauren Stratton, Naomi Meinertz, Suzanne Bartholomae

**Affiliations:** Iowa State University, Ames, Iowa, United States

## Abstract

Less than half of older adults make formal preparations to control difficult life transitions, yet planning can mitigate negative outcomes that accompany changes. To better understand multiple forms of end-of-life planning behaviors and the effect of demographic characteristics, we use data from the Understanding America Study (N=1812; aged 60+). We explored gender and marital differences in whether respondents created a will, living will, or declared a durable power of attorney for health care (DPOAHC), and who was chosen as DPOAHC (i.e., spouse, child, other relative). T-tests determined no significant gender differences in declaring a DPOAHC, creating a will, or living will. An analysis of variance (F=13.422, df=3, p<0.001) found married respondents more likely to declare a DPOAHC than separated/divorced (p<0.001) and never married respondents (p=0.002). Divorced respondents were less likely to declare a DPOAHC compared to widowed respondents (p<0.001) and widowed respondents were more likely compared to never married respondents (p=0.001). Additional analyses found the same pattern in who created a will or living will. A follow-up analysis of variance found no gender differences within marital status categories (i.e., married men versus married women). Overall, respondents who were never married were less likely to have prepared any forms of end-of-life planning. These results highlight the importance of having close relationships on the level of preparedness for end-of-life planning. Additional implications include increasing awareness about end-of-life planning for more vulnerable audiences. Future analyses will integrate findings regarding financial DPOAHC and examine other socio-demographic characteristics, such as education and race.

